# Validation and Acceptability of a Cuffless Wrist-Worn Wearable Blood Pressure Monitoring Device Among Users and Health Care Professionals: Mixed Methods Study

**DOI:** 10.2196/14706

**Published:** 2019-09-14

**Authors:** Sheikh Mohammed Shariful Islam, Susie Cartledge, Chandan Karmakar, Jonathan Charles Rawstorn, Steve F Fraser, Clara Chow, Ralph Maddison

**Affiliations:** 1 Institute for Physical Activity and Nutrition School of Exercise and Nutrition Sciences, Faculty of Health Deakin University Burwood Australia; 2 School of IT Faculty of Science, Engineering and Built Environment Deakin University Burwood Australia; 3 Charles Perkins Centre Westmead University of Sydney Sydney Australia

**Keywords:** hypertension, cardiovascular disease, wearable device, blood pressure, ambulatory blood pressure monitoring

## Abstract

**Background:**

Blood pressure (BP) is an important modifiable cardiovascular risk factor, yet its long-term monitoring remains problematic. Wearable cuffless devices enable the capture of multiple BP measures during everyday activities and could improve BP monitoring, but little is known about their validity or acceptability.

**Objective:**

This study aimed to validate a wrist-worn cuffless wearable BP device (Model T2; TMART Technologies Limited) and assess its acceptability among users and health care professionals.

**Methods:**

A mixed methods study was conducted to examine the validity and comparability of a wearable cuffless BP device against ambulatory and home devices. BP was measured simultaneously over 24 hours using wearable and ambulatory devices and over 7 days using wearable and home devices. Pearson correlation coefficients compared the degree of association between the measures, and limits of agreement (LOA; Bland-Altman plots) were generated to assess measurement bias. Semistructured interviews were conducted with users and 10 health care professionals to assess acceptability, facilitators, and barriers to using the wearable device. Interviews were audio recorded, transcribed, and analyzed.

**Results:**

A total of 9090 BP measurements were collected from 20 healthy volunteers (mean 20.3 years, SD 5.4; N=10 females). Mean (SD) systolic BP (SBP)/diastolic BP (DBP) measured using the ambulatory (24 hours), home (7 days), and wearable (7 days) devices were 126 (SD 10)/75 (SD 6) mm Hg, 112 (SD 10)/71 (SD 9) mm Hg and 125 (SD 4)/77 (SD 3) mm Hg, respectively. Mean (LOA) biases and precision between the wearable and ambulatory devices over 24 hours were 0.5 (−10.1 to 11.1) mm Hg for SBP and 2.24 (−17.6 to 13.1) mm Hg for DBP. The mean biases (LOA) and precision between the wearable and home device over 7 days were −12.7 (−28.7 to 3.4) mm Hg for SBP and −5.6 (−20.5 to 9.2) mm Hg for DBP. The wearable BP device was well accepted by participants who found the device easy to wear and use. Both participants and health care providers agreed that the wearable cuffless devices were easy to use and that they could be used to improve BP monitoring.

**Conclusions:**

Wearable BP measures compared well against a gold-standard ambulatory device, indicating potential for this user-friendly method to augment BP management, particularly by enabling long-term monitoring that could improve treatment titration and increase understanding of users’ BP response during daily activity and stressors.

## Introduction

High blood pressure (BP) or hypertension is the leading risk factor for cardiovascular disease, including myocardial infarction, stroke, and kidney disease, and accounts for 14% (10.4 million) of total deaths globally [1,2]. In 2015, an estimated 874 million people had high BP, but the disease burden is estimated to be much greater as high BP remains undetected, untreated, and uncontrolled in many individuals [2,3]. Over the last three decades, the incidence of high BP has increased worldwide and is projected to increase further, mostly because of an aging population, urbanization, reduced physical activity, and unhealthy diet [2]. High BP also leads to significant declines in productivity and economic burden and remains as a significant public health challenge.

Accurate BP measurement is essential for the diagnosis and management of hypertension; however, current measurement methods are suboptimal [4]. Measurements taken by health care professionals during medical consultations can be inaccurate because of *white coat* hypertension, and infrequent measurements may not represent typical hemodynamics [5]. Home BP measurements have been recommended by several guidelines [5-7], but it is impractical to measure BP during daily bouts of activity and at night, which has prognostic and therapeutic importance [8]. Ambulatory BP measurement devices can provide frequent valid measures across a 24-hour period and are the gold standard for clinical use, but they are not appropriate for long-term monitoring because they are intrusive, cumbersome, and costly [9].

In recent years, a number of cuffless wearable devices have emerged that enable frequent and unobtrusive BP measurement throughout the user’s usual everyday activities. Wearable cuffless BP devices are broadly defined as those worn on/attached to the body and without a pneumatic cuff, and they overcome many limitations of traditional ambulatory devices. Therefore, these devices may be suitable for collecting regular BP measurements over prolonged periods. Regular long-term monitoring could enable more comprehensive assessment of BP status and treatment adherence [10] as long as measurement accuracy meets guideline recommendations [11,12]. In a comprehensive literature review, we identified a number of commercially available cuffless, continuous, wearable BP monitoring devices [10] and selected a wrist-worn device that could be used in day-to-day life. However, before recommending the use of this device, it must be validated against standard BP measurement devices. At the same time, as these devices have only recently become available as consumer-grade products for BP measurement, little is known about the users’ and health care professionals’ perspective about their use in real life. Therefore, the primary aim of this study was to validate a wrist-worn cuffless wearable BP device against a gold-standard ambulatory BP device. Secondary aims of this study were to compare the wearable BP measurements against a home BP device and assess the acceptability of a wearable device among end users and health care professionals.

## Methods

### Study Design

A mixed methods (quantitative and qualitative) approach was used in this study.

### Participants

We recruited a convenience sample of 20 healthy volunteers via advertisements and flyers at the Deakin University Burwood campus and selected general practice clinics in Melbourne, Australia. We included adults (aged ≥18 years) with normal BP (<140/90 mm Hg) who were willing to wear an ambulatory device for 24 hours, a wearable device for 7 days, and record home BP 3 times per day for 7 days. Participants with high BP (>140/90 mm Hg), serious medical conditions, and limited mobility and those taking BP medication at baseline were excluded. In addition, we purposively recruited 10 health care professionals who manage patients with high BP and had clinical experience with a range of BP devices—including cardiologists, general practitioners, nurses, pharmacists, and exercise physiologists—to ascertain the barriers and facilitators of wearable devices and acceptability for use in their clinical practice.

### Ethics

Written informed consent was obtained from all participants at the time of enrollment. The study was approved by the Deakin University Faculty of Health Human Ethics Advisory Group (HEAG-H 135_2017).

### Data Collection and Variables

A research assistant was trained in data collection procedures, device configuration, testing, and operation for 1 week at the Deakin University. Data were collected from October 2017 to April 2018. Data regarding socioeconomic status (age, sex, education, employment, occupation, and income), self-reported comorbidities, smoking, alcohol use, cognitive function, physical activity (light, moderate, and vigorous activities/times per week for ≥15 min), diet (fruits and vegetable consumption: servings/week), and medication were collected face-to-face using a standardized questionnaire. Weight and height were measured at enrollment. Body mass index was calculated as weight in kilograms divided by height in meters squared.

### Blood Pressure Measurements

**Figure 1:**
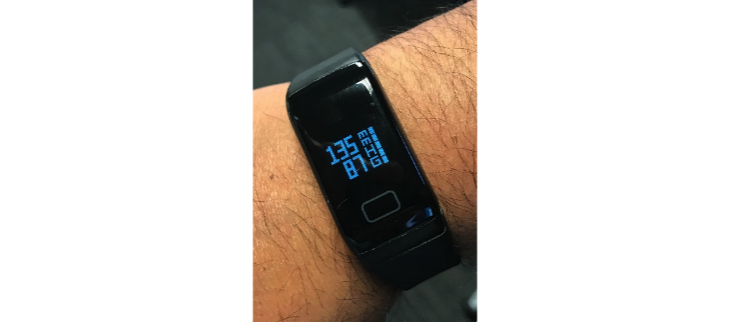
Wearable blood pressure device (T2 Mart blood pressure device).

Baseline BP was measured using an Omron automated device (Omron HEM 7121; Omron Corp) at the time of enrollment. A total of 3 baseline measures were obtained. The first measurement was discarded, and the mean of the remaining 2 readings was calculated following standard practice [13]. Starting at the time of enrollment, study participants were fitted with an ambulatory BP device (Model TM-2430; A & D Medical Corp) with appropriate cuff size, which is highly accurate and has been validated according to international standards and recommended for clinical use [14]. The ambulatory device was programmed to measure BP every 30 min during the day and every 60 min during the night for 24 hours. There were no restrictions of daily activities. Concurrently, study participants were asked to use the wearable cuffless device (Model T2; TMART Technologies Limited; Figure 1) in the nondominant hand, for example, a right-handed person was instructed to use the wearable device in the left hand. The wearable device measured BP every 60 min for 7 days. Participants were also provided with a home BP device (Omron HEM 7121) and a printed log sheet to measure and record BP 3 times per day—morning, afternoon, and evening, at consistent times self-selected by participants—for 7 days. All participants received instruction and demonstration about how to use each device at enrollment.

The wearable cuffless device was chosen as it is commercially available in Australia, is affordable (approximately Aus $50), is lightweight and easy to wear, and is waterproof and thus can be worn at all times. Participants were asked to wear the device on the wrist of their nondominant hand. This device uses MPU6500 sensors that do not require calibration before use, and it uses Bluetooth functionality to wirelessly send data to a mobile phone app (Wearfit) for storage and self-monitoring visualization. The device (minimum OS compatibility is Android 4.4 or iOS 8.0) also measured heart rate, blood oxygen saturation, sleep time, steps, mileage, and calories consumed among other features. However, these parameters were not considered in this study.

Data from the wearable and ambulatory devices were downloaded from the Wearfit app and onboard memory, respectively, after the participation period. Home BP measurements were recorded manually on a preformatted study log.

### Semistructured Interviews

Interviews were conducted with users at day 7 to determine the acceptability and usability of the wearable BP device. The interviews comprised questions about the overall experiences of using the provided BP measurement devices. Brief semistructured interviews were also conducted with health care professionals to determine acceptability as well as barriers and facilitators to real-world use. Health care professionals were briefed about the wearable BP device and how it worked. They were asked about the use of patient-reported BP data in their clinics and the future usability of wearable BP data. All interviews were audio recorded and transcribed verbatim.

### Outcomes

The primary outcome was the mean difference between wearable and ambulatory BP measurements over 24 hours. Secondary outcomes included the mean difference between wearable and home BP measurements over 7 days, barriers and facilitators of using wearable BP devices by users, and the acceptability of wearable devices among a diverse group of health care professionals.

### Data Analysis

Data were presented as mean, SD, and range. Wearable BP measurements were compared against corresponding reference ambulatory and home measurements (eg, Wearable BP device 1st measurement W1 vs Reference device 1st measurement R1; see Table 1). We manually investigated extreme values (5th, 10th, and 15th percentiles from both sides of the distribution) for all devices to check for possible measurement noise. As the data did not differ significantly, we used the full dataset from all devices in these analyses. Data were explored graphically using box plots and scatter plots. We considered subjective daily average between wearable and ambulatory devices. We also estimated daytime BP using measures recorded between 7 am and 9 pm. A *P*<.05 was considered statistically significant. Missing data were not imputed. Data analyses were performed using MATLAB 2017a software.

**Table 1:** Procedure for reference and wearable device blood pressure measurements validation.

BP^a^ measurement		Number
**Initial BP measurements^b^**		
	Take reference BP measurement (office BP)	R^c^0
	Take wearable device BP measurement	W^d^0
**Validation BP measurements for accuracy evaluation**		
	Take first reference BP measurement (mean 24-hour ambulatory BP monitoring)	R1
	Take first wearable device BP measurement (mean 24-hour wearable device)	W1
	Take second reference BP measurement (mean 7-day home BP monitoring)	R2
	Take second wearable device BP measurement (mean 7-day wearable device)	W2

^a^BP: blood pressure.

^b^Measurement R0 was not used in the evaluation of reference BP distribution and variability criteria. Measurements R0 and W0 were not used in the evaluation of the test device accuracy.

^c^R: reference blood pressure device.

^d^W: wearable blood pressure device.

We used nonparametric Mann-Whitney U tests to compare the mean (SD) of the devices. Systolic and diastolic measurement biases were calculated as reference wearable measurement. We also assessed measurement accuracy by calculating the mean absolute difference (MAD) and mean absolute percentage differences (MAPD) between the devices [14]. The MAD and MAPD were calculated as follows: MAD= (Σ^n^_i=1│_ p_i_ – y_i│_)/n and MAPD=(Σ^n^_i=1│_ 100 (p_i_ – y_i│_ y_i_)/n, where *p_i_* and *y_i_* are the average wearable and reference device measurements, respectively, and *n* is the sample size. Measurement accuracy was graded according to the following accepted clinical standards: grade A, MAD less than or equal to 5 mm Hg; grade B, MAD 5 to 6 mm Hg; grade C, MAD 6 to 7 mm Hg; and grade D, MAD greater than or equal to 7 mm Hg [15]. Relative reliability was estimated by calculating Pearson correlation coefficients to compare the degree of association [16]. Standardized Bland-Altman scatterplots and limits of agreement (LOA) were used to assess absolute reliability and the variability of measurement biases across the measurement range.

A content analysis was applied to the semistructured interview data [17]. This method is appropriate, given our aims to describe (1) usability and acceptability of the wearable device among participants and (2) acceptability of this method among health care professionals caring for people with hypertension. Once transcripts were read in their entirety, coding of main themes was performed manually, and a log was maintained in a spreadsheet form. Categories of code were developed from the data through an iterative process. Coding was initially performed separately for study participants and health care professionals to assess if there were consistent categories and themes between groups.

## Results

### Study Participants and Measurements

A total of 9090 systolic BP (SBP) and diastolic BP (DBP) data (1530 ambulatory, 6720 wearables, and 840 home devices) were analyzed. Participants’ age was 20.3 (SD 5.4) years, half were female, and mean baseline BP was 112/74 mm Hg. Additional participants’ characteristics are reported in Table 2.

**Table 2:** Characteristics of the study participants.

Characteristics		Study participants (n=20)
Male, n (%)		10 (50)
**Age (years)**		
	Mean (SD)	20.3 (5.4)
	Range	37.2-18.5
**Body mass index**		
	Mean (SD)	23.6 (3.3)
	Range	19.9-25.9
Married/living with partner, n (%)		8 (40)
Education (master’s or above), n (%)		13 (65)
Employment (fulltime), n (%)		12 (60)
**Baseline systolic BP^a^**		
	Mean (SD)	112.35 (9.79)
	Range	95-131
**Baseline diastolic BP**		
	Mean (SD)	73.75 (9.14)
	Range	47-96

^a^BP: blood pressure.

### Measurement Biases

**Figure 2:**
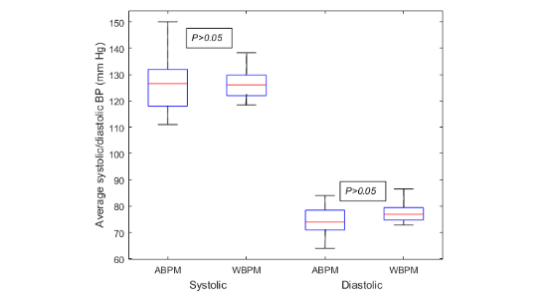
Box plot of ambulatory and wearable systolic and diastolic BP measurements. *P* value calculated using nonparametric Mann-Whitney U Test. *P*>.05 indicates the absence of systematic measurement bias between devices. ABPM: ambulatory blood pressure monitoring; BP: blood pressure; WBPM: wearable blood pressure monitoring device.

**Figure 3:**
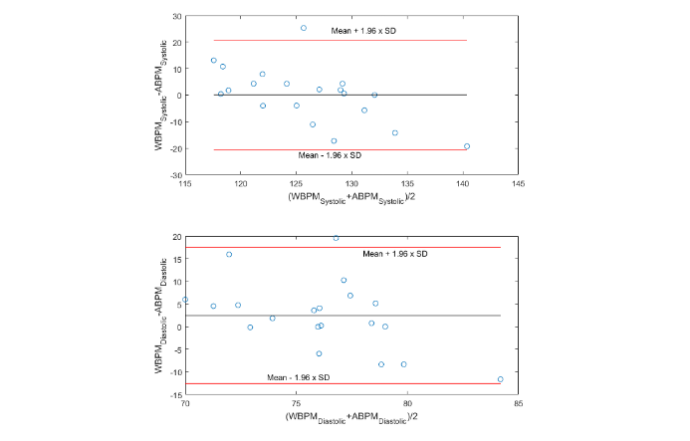
Bland-Altman plots between wearable and ambulatory blood pressure monitoring devices over 7 days. Measurement uncertainty during 24 hours of concurrent ambulatory blood pressure monitoring and wearable blood pressure monitoring. Black reference line represents mean bias, and red reference lines represent 95% limits of agreement. ABPM: ambulatory blood pressure monitoring; WBPM: wearable blood pressure monitoring device.

Table 3 summarizes BP measured across devices; BP was similar for both ambulatory and wearable devices (SBP 126 [SD 10] vs 125 [SD 5]; DBP 75 [SD 6] vs 77 [SD 9]), and there were no statistically significant differences over 24 hours (*P*>.05). The MAD between wearable and ambulatory devices over 24 hours was less than 7 mm Hg for both SBP and DBP. The average 24-hour BP data obtained using the wearable and ambulatory device showed poor relationship (SBP: r=0.16, *P*=.51; DBP: r=−0.15, *P*=.53; Multimedia Appendix 1). Mean SBP and DBP measured by ambulatory and wearable BP devices did not differ significantly (*P*>.05; Figure 2). Figure 3 shows the Bland-Altman plots comparing wearable and ambulatory devices; the mean biases (LOA) was 0.5 (−10.1 to 11.1) mm Hg for SBP and 2.24 (−17.6 to 13.1) mm Hg for DBP. The mean difference (2 SDs) were 0.08 (2 SD 20.69) in SBP and 2.46 (2 SD 15.03) in DBP.

BP values differed significantly between wearable and home devices over 7 days (*P*≤.01; see Table 3). Mean (LOA) daytime biases for wearable versus home devices were −13.9 (−33.8 to 5.9) mm Hg for SBP and −6.4 (−24.6 to 11.8) mm Hg for DBP. Daytime 7-day mean (SD) SBP and DBP were 126 (SD 6) mm Hg and 78 (SD 4) mm Hg, respectively. Moreover, 7-day MAD was greater than 7 mm Hg for SBP and less than 7 mm Hg for DBP. The mean biases (LOA) between wearable and home devices over 7 days were −12.7 (−28.7 to 3.4) mm Hg for SBP and −5.6 (−20.5 to 9.2) mm Hg for DBP. Daytime biases were similar (SBP −13.9 [−33.8 to 5.9] mm Hg; DBP −6.4 [−24.6 to 11.8] mm Hg; Multimedia Appendices 2 and 3). Similar differences were observed in the MAPD between wearable and home devices.

**Table 3:** Blood pressure values measured by different devices.

BP^a^			Mean (SD)	Range	MD^b^ (SD)	MAD^c^ (SD)	MAPD^d^ (SD)	Limits of agreement, MD (2SDs)	*P* value
**Device worn for 24 hours**									
	**Systolic BP**				**0.08 (10.56)**	**7.63 (7.09)**	**6.01 (5.42)**	**0.08 (20.69)**	**>.05^e^**
		Wearable	125 (5)	119-138					
		Ambulatory	126 (10)	111-150					
	**Diastolic BP**				**2.46 (7.67)**	**5.90 (5.34)**	**7.48 (6.45)**	**2.46 (15.03)**	**>.05^e^**
		Wearable	77 (9)	72-87					
		Ambulatory	75 (6)	64-90					
**7 days**									
	**Systolic BP**				**13.19 (8.31)**	**13.19 (8.31)**	**10.56 (6.64)**	—	**<.01^f^**
		Wearable	125 (4)	113-139					
		Home	112 (10)	85-135					
	**Diastolic BP**				**5.86 (7.62)**	**7.28 (6.21)**	**9.37 (8.04)**	**—**	**<.01^f^**
		Wearable	77 (3)	68-87					
		Home	71 (8)	50-90					

^a^BP: blood pressure.

^b^MD: mean difference.

^c^MAD: mean absolute difference.

^d^MAPD: mean absolute percentage difference.

^e^*P* for mean difference between ambulatory and wearable (24 hours) devices.

^f^*P* for mean difference between home (7 days) and wearable (7 days) devices.

### Acceptability and Barriers and Facilitators of Use

Participants were asked about the experience of using the 3 different BP devices throughout the study period. Positive and negative features of each device were clearly identified from the content analysis (Table 4). The 24-hour ambulatory device was consistently identified as being the most difficult to use and wear as it intruded into activities of daily living such as sleeping and exercising and was often uncomfortable to wear or painful during measurements. Although the home BP monitoring device was simple to use and did not interrupt daily activities, participants identified that obtaining BP measurements was reliant on them taking action and initiating a measurement. Most participants preferred the unobtrusive design and automated measurements of the wearable BP device. Although additional wearable device parameters such as heart rate and sleep quality were not formally considered in this study, participants liked being able to view these data on the device display and/or in the smartphone app. Barriers to using the wearable device included difficulty in obtaining a good fit for people with small wrists, the need for regular charging, and a motion-activated light that woke some participants during sleep.

**Table 4:** Advantages and disadvantages of using the study blood pressure devices.

Device	Advantages	Disadvantages
Home BP^a^ monitor	P^b^05: “I thought it was so quick and easy to administer, and simple to use.”	P05: “It does rely on the participant to remember to take the readings but as long as they adhere to that...it’s quite a quick process.”
24-hour ambulatory device	P08: “I suppose the positive of that [device] is that it was automatic. Um, it’s probably the only positive.”	P14: “It’s quite stressful to wear, I think it raised my blood pressure (laughs)…because you are just there, waiting every half an hour for it to go [take measurement].”
Wearable device	P13: “If you wear the watch in the morning you’re done for the day.”; P07: “You don’t notice it at all, in terms of it collecting any measurements.”	P02: “I would question…the accuracy of the BP measurement. It just didn’t seem to match up with all the...results from either of the other ones [measurements from other devices].”; P06: “When I was asleep I must have moved my wrist. And you know how it automatically lights up? It woke me up.”

^a^BP: blood pressure.

^b^P: participant.

Although both users and health care professionals identified many advantages of the wearable BP device compared with other devices, accuracy was a consistent theme identified by both participant groups. Terms such as validity, consistency, and reliability came up frequently in both groups, demonstrating the strong presence of this theme.

Most health care professionals described using and encouraging home BP monitoring for their patients. They acknowledged using patient-reported BP data when available, but they reported concerns regarding its reliability. When asked about the use of a wearable BP device by patients, all health care professionals expressed interest in this method as long as accuracy could be demonstrated. Health care professionals foresee that the benefits of a wearable device could include convenience (small size and regular automated measurements) and improved adherence to monitoring. Additional considerations for using wearable devices included the cost, data privacy, and use among vulnerable populations such as the elderly and those with English as a second language.

## Discussion

In this study, we attempted to validate, for the first time, a wearable cuffless BP device for measuring BP continuously against a gold-standard ambulatory device and against a common home BP device. Our results suggest the wearable device compared well with the gold-standard ambulatory device over 24 hours as measurement biases were within acceptable limits, but these are not sufficient on their own to recommend wearable devices as a replacement for established ambulatory devices. In contrast, wearable BP measures differed systematically from the home device over 7 days. Given the comparability of wearable and ambulatory measures, this likely suggests the home device systematically underestimated BP. The wearable device could potentially be used for long-term BP monitoring and management if their long-term validity and reliability could be established. The wearable device was acceptable to participants and health care professionals, provided validity can be ensured.

A 7-day BP measure could provide more stable assessment of BP status than current infrequent/one-off methods, but current devices are either too cumbersome (ambulatory) or impractical (home devices) for this purpose. Our findings suggest that the 24-hour data from the wearable device were consistent with its 7-day data, which might represent true BP better than the measures from a home BP device as those readings were taken only 3 times a day compared with BP data recorded hourly in a wearable device. An important assumption is that over the 7-day period, a participant’s true BP and within-participant variance could reasonably be assumed to be stable. The longer time frame allows for a sufficient number of measurements to capture the within-participant variance; however, the stability of the mean BP is less certain. The observed difference in BP between the wearable and home devices could also be because of the wearable device measuring BP during movement and daily activity, which we could not account for.

A systematic review and meta-analysis of 52 prospective studies reported that compared with usual care, self-monitoring of BP alone resulted in statistically significant improvements in SBP and DBP (−3.9 and −2.4 mm Hg, respectively) at 6 months, and that self-monitoring in combination with additional support lowered SBP from −2.1 to −8.3 mm Hg and DBP from 0.0 to −4.4 mm Hg at 12 months [18]. The unobtrusive design of the wearable device makes it well suited for assessing long-term and diurnal patterns of BP with potential prognostic significance. The wearable device used in our study is simple to set up, lightweight (23 g), waterproof, and compatible with both Android and IOS operating systems, and with a battery capacity of 80 mAh, it has a standby time of 10 days. The device can be demonstrated to the participants at the physician’s clinic during consultation. As demonstrated by our qualitative data, the device is easy to use and requires minimal technical knowledge to operate, making wearable BP devices suitable for people of all ages.

A number of wearable cuffless BP devices have become available in recent years [19-23], highlighting their potential use in clinical settings. However, most of them have only been validated for point measurements in clinical consultation settings, and longer-term validity has not been assessed. The American National Standards Institute/Association for the Advancement of Medical Instrumentation and the Institute of Electrical and Electronics Engineers wearable BP standard 2014-1708 require the MAD between test and referent devices to be less than 5 mm Hg [5] for grade A classification but does not provide guidelines for continuous BP device. In this study, we used an ambulatory device as a gold standard method to assess the validity of the wearable device. As home devices are frequently recommended to patients by health care professionals, we also compared the wearable device with a home device over 7 days. Although the use of home devices is typically limited to periodic measurements in the home during static rest, the wearable device allowed assessment of BP during daily activities and sleep. This higher-frequency measurement schedule could enable assessment of single and multiday variability in BP control. Moreover, as users can view BP on the device display and/or in a smartphone app, this type of device may help participants to better understand their BP and self-initiate discussion with clinicians during consultations when required. Thus, data obtained using wearable devices over time may allow measures of monthly or annual mean BP status, which could be used to monitor treatment effectiveness, adherence, or disease progression.

A major limitation of this study was a lack of exact time synchronization between the different devices. It would be interesting to compare the patterns and validation of BP measurements between the wearable and ambulatory devices over time. However, we have estimated the daytime wearable BP with home BP measurements by identifying wearable BP records from 7 am to 9 pm as this was representative of the time range for home device measurements. Subgroup and sensitivity analyses were not planned because of the small sample size participants. The wearable device used a combination of optical sensors and software algorithms to estimate BP; however, commercially sensitivity means detailed specifications are not available; this limits comparison with another wearable device. As our study participants all had normal BP, caution is required when generalizing the results to people with high BP. Finally, it was not possible to consider potential sources of error variance such as physiological fluctuation in BP and movement artifact during physical activity.

The capability of many wearable BP devices to wirelessly interface with mobile devices and a growing number of apps/digital platforms provides a means to (1) monitor and share BP data on a long-term basis, (2) alert participants to key changes in BP, and (3) help physicians to understand treatment adherence and efficacy and make appropriate adjustments. As artificial intelligence approaches become more sophisticated, it could be possible to automate data processing and synthesis, which will help to streamline the BP monitoring workflow for time-limited physicians. Further work is needed to better understand if this wearable device can identify nighttime dipping and early morning surges in BP, which provide important clinical information about hemodynamics control. Future research into validating wearable devices in clinical populations with time-stamped data shared with patients and clinicians in a meaningful way to support clinical decision making is warranted.

In conclusion, wearable BP device measures compared well against a gold-standard ambulatory device measure. Participants found the device simple and easy to wear and use. Our findings indicate potential for this method to augment BP management, particularly by enabling long-term monitoring that could improve treatment titration and increase understanding of users’ BP response during daily activity and stressors. The streamlined design and operation of wearable BP devices can offer numerous advantages compared with traditional ambulatory and home devices; however, measurement validity is a critical requirement. As this particular device did not meet established validity criteria, it cannot be recommended as a replacement for gold-standard ambulatory devices.

## Appendix

Multimedia Appendix 1 Relationships between ambulatory blood pressure monitoring and wearable blood pressure monitoring during 24 hours of concurrent monitoring.

Multimedia Appendix 2 Bland-Altman plots between wearable blood pressure monitoring (WBPM) and ambulatory blood pressure monitoring (ABPM) devices (24 hours). Measurement uncertainty during 24 hours of concurrent ABPM and WBPM. Black reference line represents mean bias, and red reference lines represent 95% limits of agreement.

Multimedia Appendix 3 Scatterplots of systolic BP versus diastolic BP for wearable and ambulatory devices. BP: blood pressure.

## Abbreviations

BP: blood pressure
DBP: diastolic blood pressure
IPAN: Institute for Physical Activity and Nutrition
LOA: limits of agreement
MAD: mean absolute difference
MAPD: mean absolute percentage difference
SBP: systolic blood pressure
