# Supplementary material for: Validation and Acceptability of a Cuffless Wrist-Worn Wearable Blood Pressure Monitoring Device Among Users and Health Care Professionals: Mixed Methods Study
Source: JMIR Mhealth Uhealth. 2019 Sep 14;7(10):e14706. doi: 10.2196/14706 (PMC6827985; doi:10.2196/14706)

Figure S1. Relationships between ambulatory (ABPM) and wearable (WBPM) blood pressures during 24 hours of concurrent monitoring.

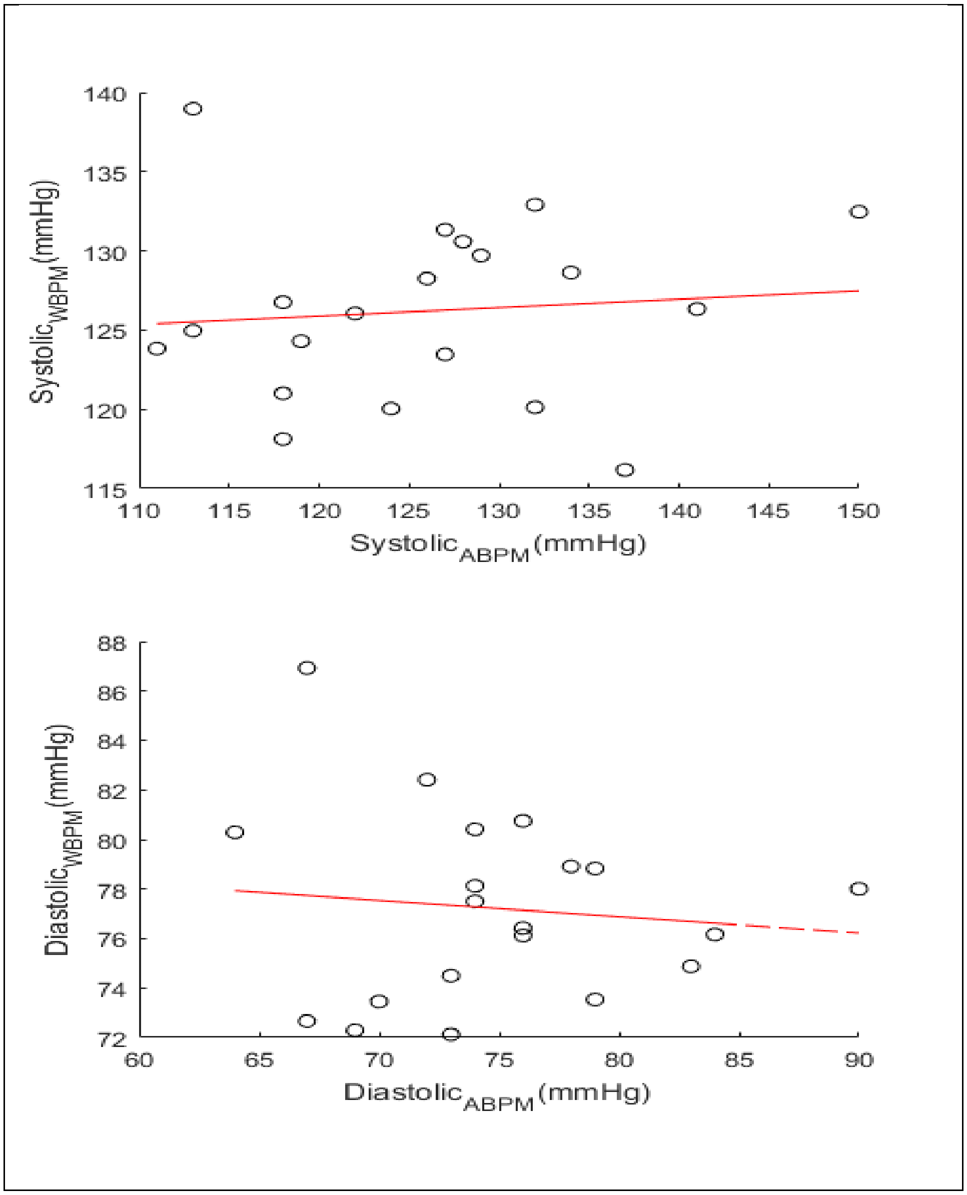

Supplement: Multimedia Appendix 1 [file mhealth_v7i10e14706_app1.pdf]
