# Supplementary material for: Validation and Acceptability of a Cuffless Wrist-Worn Wearable Blood Pressure Monitoring Device Among Users and Health Care Professionals: Mixed Methods Study
Source: JMIR Mhealth Uhealth. 2019 Sep 14;7(10):e14706. doi: 10.2196/14706 (PMC6827985; doi:10.2196/14706)
Supplement: Multimedia Appendix 2 [file mhealth_v7i10e14706_app2.pdf]

Figure S2. Bland-Altman Plots between wearable and ambulatory blood pressure monitoring devices (24-hours).

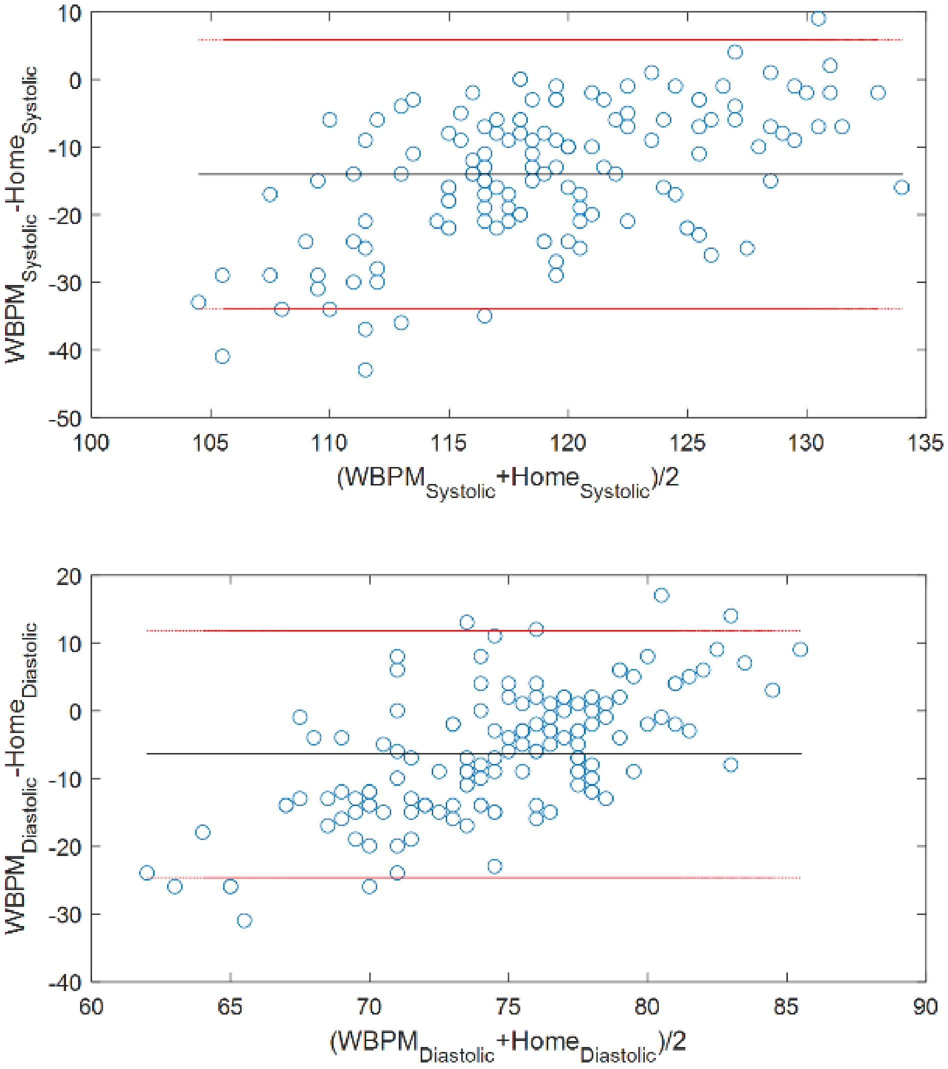

Measurement uncertainty during 24 hours of concurrent ambulatory (ABPM) and wearable (WBPM) blood pressure monitoring. Black reference line = mean bias, red reference lines = 95% limits of agreement.
