# Supplementary figures and images for: Validation and Acceptability of a Cuffless Wrist-Worn Wearable Blood Pressure Monitoring Device Among Users and Health Care Professionals: Mixed Methods Study
Source: JMIR Mhealth Uhealth. 2019 Sep 14;7(10):e14706. doi: 10.2196/14706 (PMC6827985; doi:10.2196/14706)

Figure S3: Scatterplots of SBP vs DBP for Wearable and Ambulatory devices

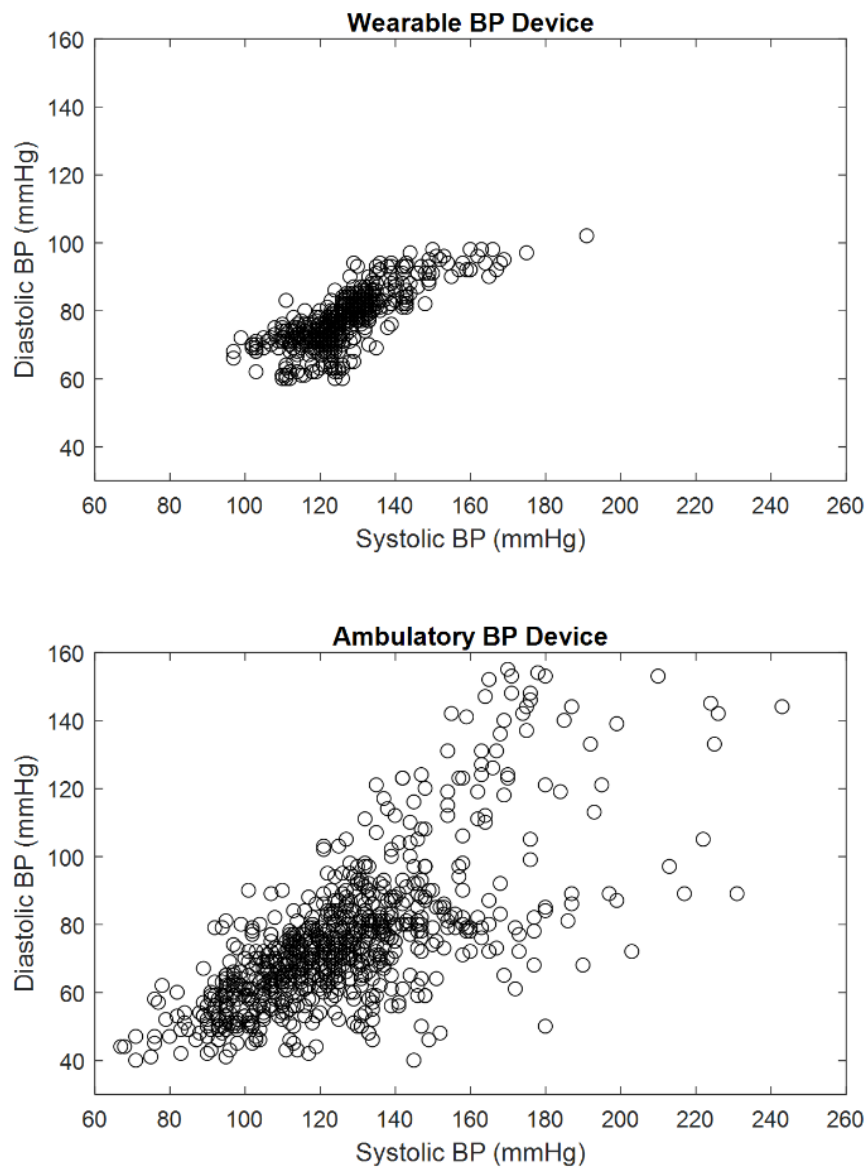

Supplement: Multimedia Appendix 3 [file mhealth_v7i10e14706_app3.pdf]
